# Chemical Composition, Enantiomeric Distribution, and Sensory Evaluation of the Essential Oils Distilled from the Ecuadorian Species *Myrcianthes myrsinoides* (Kunth) Grifo and *Myrcia mollis* (Kunth) DC. (Myrtaceae)

**DOI:** 10.3390/plants8110511

**Published:** 2019-11-15

**Authors:** Mayra Montalván, Manuel Alejandro Peñafiel, Jorge Ramírez, Nixon Cumbicus, Nicole Bec, Christian Larroque, Carlo Bicchi, Gianluca Gilardoni

**Affiliations:** 1 Departamento de Química y Ciencias Exactas, Universidad Técnica Particular de Loja (UTPL), Calle M. Champagnat s/n, Loja 1101608, Ecuador; mayste_95@hotmail.com (M.M.); mapenafiel3@gmail.com (M.A.P.); jyramirez@utpl.edu.ec (J.R.); nlcumbicus@utpl.edu.ec (N.C.); 2 Institute for Regenerative Medicine and Biotherapy (IRBM), Centre Hospitalier Universitaire de Montpellier, Inserm U1183, 34295 Montpellier, France; nicole.bec@inserm.fr; 3 Supportive Care Unit, Institut du Cancer de Montpellier (ICM), 34298 Montpellier, France; cjlarroque@gmail.com; 4 Dipartimento di Scienza e Tecnologia del Farmaco, Università degli Studi di Torino, Via P. Giuria 9, 10125 Torino, Italy; carlo.bicchi@unito.it

**Keywords:** *Myrcianthes myrsinoides*, *Myrcia mollis*, essential oil, GC-O, AEDA, AChE, BChE, Ecuador

## Abstract

The essential oils of *Myrcianthes myrsinoides* and *Myrcia mollis*, belonging to the Myrtaceae family, were obtained by steam distillation. They were analyzed by gas chromatography-mass spectrometry (GC-MS), gas chromatography-flame ionization detector (GC-FID), enantioselective gas chromatography, and gas chromatography-olfactometry (GC-O). A total of 58 compounds for *Myrcianthes myrsinoides* essential oil (EO) and 22 compounds for *Myrcia mollis* EO were identified and quantified by GC-MS with apolar and polar columns (including undetermined components). Major compounds (>5.0%) were limonene (5.3%–5.2%), 1,8-cineole (10.4%–11.6%), (*Z*)-caryophyllene (16.6%–16.8%), *trans*-calamenene (15.9%–14.6%), and spathulenol (6.2%–6.5%). The enantiomeric excess of eight chiral constituents was determined, being (+)-limonene and (+)-germacrene D enantiomerically pure. Eight components were identified as determinant in the aromatic profile: α-pinene, β-pinene, (+)-limonene, γ-terpinene, terpinolene, linalool, β-elemene and spathulenol. For *M. mollis*, the major compounds (>5.0%) were α-pinene (29.2%–27.7%), β-pinene (31.3%–30.0%), myrcene (5.0%–5.2%), 1,8-cineole (8.5%–8.7%), and linalool (7.7%–8.2%). The enantiomeric excess of five chiral constituents was determined, with (*S*)-α-pinene and (+)-germacrene D enantiomerically pure. The metabolites β-pinene, 1,8-cineole, γ-terpinene, terpinolene, linalool, and (*E*)-β-caryophyllene were mainly responsible for the aroma of the EO. Finally, the *M. myrsinoides* essential oil has an inhibitory activity for cholinesterase enzymes (IC_50_ of 78.6 μg/mL and 18.4 μg/mL vs. acethylcholinesterase (AChE) and butyrylcholinesterase (BChE) respectively). This activity is of interest to treat Alzheimer’s disease.

## 1. Introduction

Ecuador is a country rich in aromatic and medicinal plants, distributed in its different regions. The province of Loja, the place of collection of the two investigated species, lies in the Sierra [1]. Myrtaceae, Asteraceae, Anacardiaceae, Apiaceae, Lauraceae, and Rutaceae are families including a large number of essential oil-producing species [2].

The Myrtaceae family consists of woody plants, ranging from shrubs to tall trees. The species belonging to this family have multiple traditional uses, such as food, construction wood, and a source of vegetable oils. Moreover, many are traditionally used for their therapeutic effects, such as antipyretic, sedative, antifungal, antibacterial, anti-inflammatory, and hypoglycemic [3]. In Ecuador, the Myrtaceae family accounts for 83 species, of which 9 are endemic. Among these species, 9 belong to the genus *Myrcianthes* (of which 1 is endemic) and 10 to the genus *Myrcia* (of which 1 is endemic) [4]. *Myrcianthes myrsinoides* and *Myrcia mollis* are native species of this family.

The two species were selected to be presented in the same work due to the apparently similar aroma of the essential oils (EOs). In fact, during a previous qualitative sensory evaluation, the two volatile fractions were perceived by the same panelists involved in the subsequent GC-olfactometry (GC-O) analysis, who considered the aroma “discernable but quite similar”. This statement, together with fact that the plants belong to the same botanical family, induced the authors to conduct a parallel study.

*Myrcianthes myrsinoides* (Kunth) Grifo is a shrub or tree, with aromatic, small, and oblong leaves, found between 2000 and 3500 m above sea level. It is a native species described in the Ecuadorian Andean region, in the provinces of Azuay, Bolívar, Cañar, Chimborazo, Imbabura, Loja, Pichincha, and Tungurahua. It is known by several synonyms, such as *Eugenia myrsinoides* (Kunth) Burret ex Diels; *Eugenia ternifolia* O. Berg; *Eugenia triquetra* O. Berg; *Eugenia triquetra* var. *aequatorialis* O. Berg; *Myrteola myrsinoides* (Kunth) O. Berg; and *Myrtus myrsinoides* (Kunth) [5]. Its traditional name is Arrayán, and it is used in Ecuadorian traditional medicine for the treatment of toothache [6]. Its chloroform extract, containing phenolic and terpenic metabolites, has been studied for its hypoglycemic, antibacterial, and antioxidant activities, while no data are reported concerning its essential oil [3,4,7].

*Myrcia mollis* (Kunth) DC., also known as *Myrtus mollis* Kunth [5], is a native tree growing between 500 to 2000 m above sea level in the provinces of Azuay, Loja, and Napo. Its common name is Geberber, and its fruits are edible [4]. To the best of the authors’ knowledge, at present no data on its chemical composition and/or biological activity have been reported in the literature.

As part of a project aiming to valorize Ecuadorian spontaneous flora [8,9,10,11,12,13,14,15,16,17,18,19,20,21,22,23,24,25], the chemical composition and sensory profile of *Myrcianthes myrsinoides* (Kunth) Grifo and *Myrcia mollis* (Kunth) DC essential oils (EOs) are here reported for the first time. Gas chromatography mass spectrometry (GC-MS) and enantioselective GC-MS were used for the quali-quantitative analysis, and GC-olfactometry (GC-O) to evaluate the odor active compounds. Furthermore, both essential oils were tested to evaluate their inhibitory activity on acetylcholinesterase (AChE) and butyrylcholinesterase (BChE), two enzymes important as pharmacological targets in the design of drugs active against neurodegenerative diseases such as Alzheimer’s disease [26]. No relationships exist between the traditional use of these plants and Alzheimer’s disease, however our interest in AChE and BChE inhibition resides in the determination of uncommon biological activities for EOs, in order to expand their use and knowledge. In fact, most EOs are known to be antibacterial or antifungal products, what rarely leads to concrete pharmaceutical applications. The inhibition of cholinesterases represents an uncommon biological activity for EOs, of which few interesting examples have been described in literature so far [25,26].

## 2. Results

### 2.1. Chemical Analysis

The essential oil of both species was analyzed by GC-MS and gas chromatography-flame ionization detector (GC-FID), with an apolar DB-5ms (5% phenyl-dimethylpolysiloxane) column and a polar HP-INNOWax (polyethylene glycol) column. The results are reported in Table 1 and Table 2 and show that *M. myrosinoides* EO mainly consists of sesquiterpenoids (66.8%–69.2%), and *M. mollis* EO is based on monoterpenoids (88.7%–90.6%).

A total of 58 (DB-5ms) and 52 (HP-INNOWax) compounds were detected and quantified in the essential oil of *M. myrsinoides*, with sesquiterpene hydrocarbons accounting for the main fraction (46.6%–50.0%). Major compounds (>1.0%), according to the elution order, were α-pinene (2.5%), β-pinene (1.5%), α-phellandrene (1.0%–1.1%), *p*-cymene (1.2%–1.3%), limonene (5.2%–5.3%), 1,8-cineole (10.4%–11.6%), linalool (1.4%–1.6%), α-terpineol (2.0%), α-cubebene (1.4%), α-copaene (2.1%–2.2%), (*Z*)-caryophyllene (16.6%–16.8%), α-humulene (1.9%–2.0%), *cis*-muurola-4(14),5-diene (2.6%), germacrene D (2.0%–2.1%), β-selinene (1.1%–1.9%), *trans*-calamenene (14.6%–15.9%), *trans*-cadina-1,4-diene (3.5%), germacrene B (1.2%), spathulenol (6.2%–6.5%), 1-*epi*-cubebol (1.0%–1.3%), and cubenol (1.1%–1.7%). A standard deviation of less than 5% was obtained between the percentages of each analytes with both columns. Among the detected components, five were undetermined and are omitted from the table.

Regarding the *M. mollis* EO, 22 (DB-5ms) and 21 (HP-INNOWax) constituents were detected and quantified, major compounds (>1.0%) being: α-pinene (27.7%–29.2%), β-pinene (30.0%–31.3%), myrcene (5.0%–5.2%), limonene (4.6%–4.7%), 1,8-cineole (8.5%–8.7%), γ-terpinene (1.4%–1.5%), linalool (7.7%–8.2%), α-ylangene (0.9%–1.1%), (*E*)-β-caryophyllene (2.3%–2.7%), and δ-cadinene (1.2%–2.1%). A standard deviation below 5% was obtained between the percentages of each analyte on both columns. Four detected components were undetermined and are omitted from the table.

The undetermined compounds with a molecular weight of 204 or 220 are most probably hydrocarbon or oxygenated sesquiterpenoids. Their amount accounted for 0.2%–2.2% in the *M. myrosinoides* essential EO and 0.4%–3.2% in the *M. mollis* EO.

### 2.2. Enantioselective Analysis

The distribution of the enantiomeric pairs in both species’ essential oil was determined with two capillary columns coated with a chiral selector: diethyl terbutylsilyl-β-cyclodextrin and diacetyl terbutylsilyl-β-cyclodextrin [38,39].

In *M. myrsinoides* EO, two chiral constituents, (+)-limonene and (+)-germacrene D, are baseline separable one another only with the first chiral selector, where they resulted to be enantiomerically pure. The enantiomeric distribution and enantiomeric excess were calculated, and the results are shown in Table 3.

The two above chiral columns were used to measure the enantiomeric distribution and enantiomeric excess of five enantiomeric compounds in the *M. mollis* essential oil. α-Pinene, germacrene D, and α-thujene were only separated by the diethyl terbutylsilyl-β-cyclodextrin column. The results are reported in Table 4.

### 2.3. Sensory Evaluation

**Figure 1:**
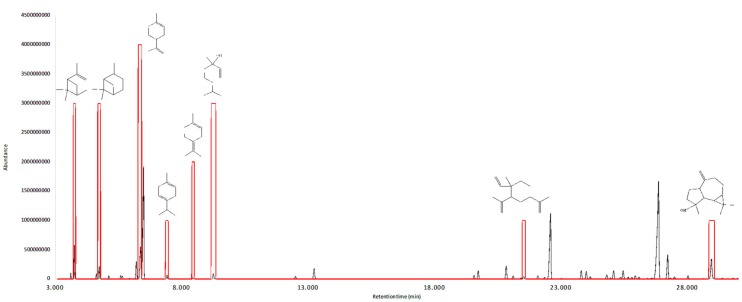
Gas chromatogram (black line) vs. aromagram (red line) of the essential oil from *M. myrsinoides.*

**Figure 2:**
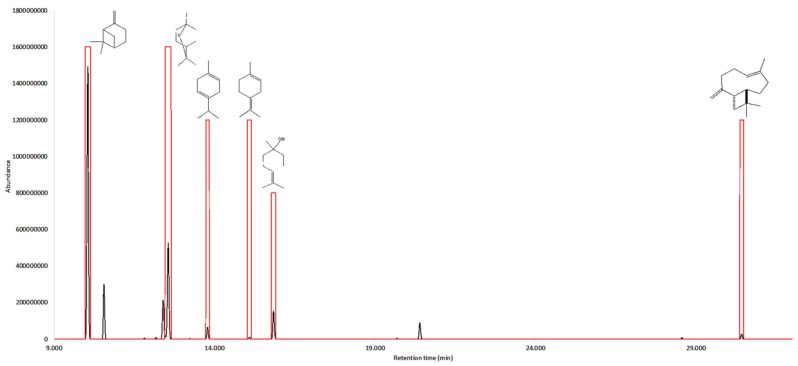
Gas chromatogram (black line) vs. aromagram (red line) of the essential oil from *M. mollis.*

The olfactive active compounds were estimated in each of the two investigated essential oils by GC-O. Table 5 reports their linear retention indices and the corresponding sensory description. In addition, an aromagram was constructed and flavor dilution factors (FD) measured by aroma extract dilution analysis (AEDA) [40], which can be visualized in Figure 1 and Figure 2.

### 2.4. Biological Activity

The inhibitory activity of the two investigated EOs were then tested on two cholinesterase enzymes: AChE and BChE. Only *M. myrsinoides* presented an inhibitory activity for the investigated enzymes, with an IC_50_ of 78.6 μg/mL for acetylcholinesterase and of 18.4 μg/mL for butyrylcholinesterase. The IC_50_ value for the *M. mollis* EO was > 50 μg/mL for both enzymes. The IC_50_ of donepezil, the positive control, was 0.04 μg/mL for AChE and 3.6 μg/mL for BChE.

## 3. Discussion

The essential oils of the two investigated species shared 13 compounds, mainly monoterpene hydrocarbons: α-thujene, α-pinene, β-pinene, myrcene, α-terpinene, *p*-cymene, limonene, 1,8-cineole, (*E*)-β-ocimene, γ-terpinene, terpinolene, linalool, and germacrene D. Monoterpenes and sesquiterpenes are known to be secondary metabolites characteristic of the Myrtaceae family EOs [7]. The genus *Myrcia* is in general characterized by (*E*)-caryophyllene, myrcene, and α-pinene as major components [41], while the genus *Myrcianthes* mainly contains 1,8-cineole, β-caryophyllene, limonene, α-pinene, carvone, and linalool [7].

GC-MS-FID analysis led to the quantification of more than 90% of the EO components for both species. Quantitation was based on the determination of the relative response factor (RRF) according to the combustion enthalpy [42]. This method assumes that the RRF depends on the elemental composition of the molecules, so that compounds with the same molecular formula and number of aromatic rings have the same RRF [43]. The present study constitutes one of the first applications of this method with external calibration, using isopropyl caproate as a calibration standard and *n*-nonane as internal standard.

Enantioselective GC analysis [44] investigated the enantiomeric ratio and excess of eight chiral components (α-thujene, α-pinene, β-pinene, sabinene, 4-terpineol, α-phellandrene limonene, and germacrene D) in both essential oils. In *Myrcianthes myrsinoides* EO, (+)-limonene and (+)-germacrene D were present in the enantiomeric pure form, while in *Myrcia mollis* EO, (*S*)-α-pinene and (+)-germacrene D were enantiomerically pure.

The sensory description of each odorant was determined by two trained panelists and resulting descriptors were consistent with the data reported in the literature. These results derived from the evaluation of two panelists are indeed not exhaustive, from a statistical point of view, to describe correctly the aromatic EO profile. However, they can be considered a contribution useful to justify the similar aroma for two chemically different products. A distinctive woody note was described for α-pinene and β-pinene [16], an herbal note for γ-terpinene, a plastic predominant odor for terpinolene, and a floral note for linalool. With *M. myrsinoides* EO, limonene was perceived with a flavor dilution factor (FD) of 16, while in the *M. mollis* EO, β-pinene and 1,8-cineole presented a FD of 8. FD were determined by aroma extract dilution analysis (AEDA), which allows a reliable qualitative and quantitative odor analysis of each analyte eluting from the chromatographic column to be obtained [15]. In this study, we can observe that the chemical analysis, showing a quite different composition, should deny the previous sensory statement on the similar aroma for the two oils. However, the GC-O evaluation indicates that *M. myrsinoides* and *M. mollis* share four main odorants (α-pinene, γ-terpinene, terpinolene, and linalool). This fact fully justifies the similar odor perception and demonstrates that GC-O is actually the main technique to deeply investigate the aromatic profile of an odorous mixture.

The biological roles of AChE and BChE are different. The former plays a fundamental role in the human nervous system, when hydrolyzing acetylcholine, a neurotransmitter of cholinergic synapses, allowing the restoration of the functionality of the postsynaptic terminals [26]. The latter does not have a well-defined physiological function but is known to act as an endogenous suppressant of anticholinergic compounds by hydrolysis of hydrophobic and hydrophilic esters [45]. The results obtained with the biological activity tests for the *M. myrsinoides* EO indicated an average maximum inhibitory concentration of 78.6 μg/mL anti-AChE and 18.4 μg/mL anti-BChE, i.e., values close to those reported for other essential oils rich in pinene isomers [46]. Furthermore, compared to the positive control, *M. myrsinoides* essential oil was almost inactive for AChE and 4.8 less active for BChE.

However, the much higher activity against BChE than AChE is consistent with a similar case reported by the authors in a recent publication [25], where the difference was hypothetically attributed to a possible selective inhibitory mechanism. On the other hand, the lack of activity for *M. mollis* EO, which presents a more important monoterpene composition, could be related to the intriguing possibility that the opposite enantiomeric excess of some chiral constituents, such as α-pinene and limonene, is responsible for the different activity.

## 4. Materials and Methods

### 4.1. Materials and Methods

The chemical, enantioselective, and GC-O analyses were carried out with an Agilent Technologies GC-MS system consisting of a 6890N gas chromatograph with a 7683 autoinjector combined with a 5973 INERT mass spectrometric detector (Santa Clara, CA, USA) and a flame ionization detector (FID). The instrument was also equipped with a Gerstel ODP 3 sniffing port (Gerstel GmbH Co., Mülheim an der Ruhr, Germany).

The mass spectrometer detector operated in SCAN mode (40–350 *m/z*), with an electron ionization source at 70 eV.

The qualitative and quantitative analyses were run with both an apolar and a polar capillary column. The apolar column was 5% phenyl methylpolysiloxane stationary phase (DB-5ms from Agilent Technologies, 30 m long, 0.25 mm internal diameter, and 0.25 μm film thickness); the polar column was a polyethylene glycol stationary phase (HP-INNOWax from Agilent Technologies, 30 m × 0.25 mm × 0.25 μm).

The enantioselective analyses were performed with two enantioselective capillary columns, using both a 30% diethyl tertbutylsilyl-β-cyclodextrin in PS-086 and a diacetyl tertbutylsilyl-β-cyclodextrin in OV-1701 as chiral stationary phases as chiral selectors. Both columns were 25 m × 250 μm × 0.25 μm, and were purchased from Mega, MI, Italy.

The GC-O analyses were run with the above DB-5ms column at the exit of which a flow splitting of 50% between detector (FID) and sniffing port was applied. Carrier gas for all analysis was helium GC purity (Indura, Guayaquil, Ecuador).

The enzyme inhibition tests were carried out with a Varioskan Flash detection system, purchased from Thermo Scientific (Waltham, MA, USA).

All solvents, alkanes and internal standard were analytical grade (purity > 99%), from Sigma-Aldrich (Saint Louis, MO, USA). The calibration standard was isopropyl caproate, obtained in the authors’ laboratory by synthesis and purified to 98.8% (GC-FID purity).

### 4.2. Plant Material

*Myrcianthes myrsinoides* and *Myrcia mollis* were collected in the canton of Gonzanamá, province of Loja, under the permission of the Ecuadorian Ministry of the Environment (MAE-DNB-CM-2016-0048). The place of collection corresponded to the following coordinates: 4° 5′ 28.03″ S and 79° 30′ 27.27″ W, at the height of 1820 m. The species were identified by one of the authors (N.C.); a botanical sample of *M. myrsinoides* and *M. mollis* was prepared and deposited at the herbarium of the Universidad Técnica Particular de Loja (UTPL), with voucher codes HUTPL-13742 and HUTPL-13743, respectively.

### 4.3. Distillation of the Essential Oil

The essential oils of both plants were obtained by steam distillation for 4 hours, using a Clevenger-type stainless steel apparatus. The fresh *M. myrsinoides* plant material (leaves) was distilled in four repetitions, two with 2.2 kg and two with 0.6 kg; the yield was 0.3% ± 0.01% (w/w). The fresh *M. mollis* leaves were also distilled in four repetitions of 1.9 kg each, obtaining a yield of 0.2% ± 0.02% (w/w). The EOs were immediately dried on anhydrous sodium sulfate and stored in the dark at −4 °C.

### 4.4. Chemical Analyses

The analytical samples were prepared by diluting an exactly weighed amount of essential oil (corresponding to 10 μL) with 1 mL of internal standard solution, previously prepared by diluting 0.7 mg of *n*-nonane to a total volume of 10 mL with cyclohexane. Such a preparation was repeated for each EO of the two species. These samples were directly used for the qualitative, quantitative, and enantioselective analyses.

*GC-O analyses*: two concentrated EO samples were prepared by diluting 30 μL of *M. myrsinoides* EO in 500 μL of cyclohexane and 10 μL of *M. mollis* EO in 1 mL of the same solvent.

*Qualitative analysis:* the GC-MS analyses of *M. myrsinoides* EO for both DB-5ms and HP INNOWax columns were carried out under the following conditions: temperature program from 60 °C (5 min) to 180 °C at 3 °C/min then to 250 °C (5 min) at 15 °C/min. The injector operated in split mode, with a ratio of 40:1; injection volume of 1 μL, and temperature of 250 °C; helium flow rate: 1 ml/min. The GC-MS qualitative analyses of *M. mollis* EO for both DB-5ms and HP INNOWax columns were carried out under the following conditions: temperature program from 60 °C (5 min) to 165 °C at 3 °C/min then to 250 °C (5 min) at 15 °C/min. The injector operated in split mode, with a ratio of 50:1; injection volume of 1 μL and temperature of 250 °C; helium flow rate: 1 ml/min. Additionally, a mixture of *n*-alkanes (C_9_–C_25_) was injected under the same conditions to determine the linear retention indices (LRIs).

*Quantitative analysis:* all samples of *M. mollis* and *M. myrsinoides* EOs, prepared as previously described, were analyzed by GC-FID with both columns, under the same instrumental conditions described for qualitative analyses. Four calibration curves were obtained, injecting six dilutions of isopropyl caproate (calibration standard) and *n*-nonane (internal standard) for each EO in both columns. The dilutions were obtained by diluting 0.8 mg, 1.8 mg, 4.1 mg, 8.3 mg, 16.9 mg, and 34.5 mg of isopropyl caproate and an exactly weighed amount of 7.6–7.8 mg of *n*-nonane to 10 mL with cyclohexane. All calibration curves achieved a R^2^ > 0.999.

### 4.5. Enantioselective Analyses

The enantioselective analyses were carried out by GC-MS under the following temperature program for both EOs: from 60 °C (5 min) to 220 °C (5 min) at 2 °C/min. The injector operated in split mode, with a ratio of 40:1; injection volume of 1 μL, and temperature 220 °C. A mixture of *n*-alkanes (C_9_–C_25_) was injected under the same conditions as for conventional analysis to determine LRIs.

The identification of each enantiomer was achieved by injecting a series of enantiomerically pure standards, available from one of the authors (C.B.).

### 4.6. AEDA Analysis

The GC-O analyses were performed according to the AEDA method for both species, by injecting 2 μL of each sample, prepared as described in Section 4.4, in a sequence of increasing dilutions until no odor was perceived by all the panelists. The dilutions were obtained by operating on the split values of 5:1, 10:1, 20:1, 40:1, and 80:1 for both species, and up to 160:1 for *M. myrsinoides* EO.

The following temperature program was applied: 40 °C (1 min) to 280 °C (10 min) at 12 °C/min. Helium flowrate: 2 mL/min.

Two operators ran the olfactory analysis, perceiving the odors without visualizing the chromatogram and describing the perceived aroma of each analyte eluting at the sniffing port. The adopted acceptance criteria implied that a perception, to be accepted, need to be detected by at least one panelist in two following dilutions or by both panelists in a single dilution. The results allowed the construction of an aromagram over the chromatogram, based on the LRI and the FD of each odor described.

### 4.7. Biological Activity

The activities of cholinesterase (ChE) were evaluated by following a colorimetric protocol adapted from the literature [46,47,48]. The catalyst efficiently hydrolyzes acetylthiocholine (ACh), the sulfur analogue of the natural substrate of these enzymes. After hydrolysis, this substrate analogue produces acetate ion and thiocoline. Thiocoline reacts with the highly reactive 5,5’-dithiobis-(2-nitrobenzoic acid) ion (DTNB) to give a yellow color, which can quantitatively be monitored by measuring its spectrophotometric absorption at 412 nm. The inhibition assay volume contained 200 μL of phosphate buffered saline (pH 7.4), DNTB (1.5 mM) and test sample in DMSO (1% v/v). The assay was carried out on *Electrophorus electricus* acetylcholinesterase and equine serum butyrylcholinesterase that were both dissolved in PBS pH 7.4 and used at 25 mU/mL. After 10 minutes of preincubation, the substrate acetylthiocholine iodide (1.5 mM) was added to start the reaction. Multiple 96-well microliter sites were read in the detection system during 30 min of incubation at 30 °C. All measurements were carried out in triplicate. When possible, the IC_50_ values were calculated using the GNUPLOT package online (www.ic50.tk, www.gnuplot.info). Donepezil was used as reference ChE inhibitor and showed an IC_50_ = 100 nM for AChE and 8500 nM for BChE. With this assay, the possibility of false positive inhibition results previously described for high concentration (> 100 μg/mL) of amine or aldehyde compounds cannot be excluded [25].

## 5. Conclusions

In this study, the chemical, enantiomeric, and sensory profiles of two EOs were investigated for the first time. The EO of *M. myrsinoides* was established to consist mainly of sesquiterpenoids, while in the *M. mollis* EO, monoterpenoids prevail. Despite the different chemical compositions, the similarity in the AEDA evaluation could justify the similarity of the two aromas.

Furthermore, the EO of *M. myrsinoides* was determined to be a weak selective inhibitor of BChE, with an inhibitory activity hypothetically attributable to a chirality dependent mechanism of monoterpenes (IC_50_ of 78.6 μg/mL and 18.4 μg/mL vs. AChE and BChE, respectively).

## Figures and Tables

**Table 1:** Chemical composition of the essential oil of *M. myrsinoides* in DB-5ms and HP-INNOWax columns.

N.	Compound	LRI	DB-5ms LRI_r_ [27]	%^1^	σ^2^	LRI	HP-INNOWax LRI_r_	%^1^	σ^2^
1	α-thujene	913	924	0.4	0.17	1022	1027 [28]	0.4	0.15
2	α-pinene	919	932	2.5	1.18	1017	1025 [28]	2.5	1.00
3	sabinene	960	969	0.5	0.36	1118	1122 [28]	0.5	0.33
4	β-pinene	966	974	1.5	0.71	1105	1110 [28]	1.5	0.60
5	myrcene	984	988	0.6	0.37	1164	1161 [29]	0.5	0.34
6	α-phellandrene	1003	1002	0.5	0.30	1161	1168 [28]	0.5	0.32
7	δ-3-carene	1005	1008	0.4	0.11	1144	1147 [28]	0.3	0.10
8	α-terpinene	1012	1014	0.1	0.03	1176	1178 [28]	0.1	0.03
9	*p*-cymene	1019	1020	1.2	0.62	1269	1270 [28]	1.3	0.63
10	limonene	1024	1024	5.3	2.58	1197	1198 [28]	5.2	2.42
11	1,8-cineole	1027	1026	10.4	7.88	1204	1211 [28]	11.6	7.58
12	(*E*)-β-ocimene	1041	1044	0.1	0.01	1252	1250 [28]	0.1	0.02
13	γ-terpinene	1051	1054	0.5	0.11	1243	1245 [28]	0.5	0.10
14	terpinolene	1079	1086	0.3	0.11	1280	1282 [28]	0.3	0.09
15	linalool	1100	1095	1.4	0.84	1553	1554 [29]	1.6	0.89
16	4-terpineol	1174	1174	0.6	0.31	1595	1601 [28]	0.4	0.32
17	α-terpineol	1191	1186	2.0	1.09	-	-	-	-
18	*p*-mentha-1,4-dien-7-ol	1334	1325	0.6	0.33	-	-	-	-
19	α-cubebene	1338	1348	1.4	0.34	1450	1460 [28]	1.4	0.31
20	α-copaene	1363	1374	2.1	0.33	1481	1491 [28]	2.2	0.32
21	β-bourbonene	1370	1384	0.6	0.08	1508	1507 [30]	0.7	0.10
22	β-cubebene	1377	1387	0.4	0.14	1530	1542 [28]	0.5	0.13
23	β-elemene	1380	1389	0.7	0.18	-	-	-	-
24	α-gurjunene	1393	1409	0.9	0.31	1519	1529 [28]	0.9	0.30
25	methyleugenol	1399	1403	0.4	0.14	2019	2006 [28]	0.6	0.10
26	(*Z*)-caryophyllene	1405	1408	16.6	6.29	1585	1588 [28]	16.8	5.29
27	β-copaene	1415	1430	0.4	0.15	1579	1580 [28]	0.8	0.16
28	α-humulene	1440	1452	1.9	0.66	1655	1667 [28]	2.0	0.58
29	aromadendrene	1443	1439	0.6	0.17	1629	1620 [28]	0.8	0.15
30	*allo*-aromadendrene	1463	1458	0.3	0.07	1617	1630 [31]	1.8	1.32
31	*cis*-muurola-4(14),5-diene	1466	1465	2.6	0.85	1648	1643 [28]	2.6	0.73
32	β-chamigrene	1473	1476	0.5	0.08	1708	1724 [28]	0.5	0.08
33	germacrene D	1475	1480	2.1	0.67	1697	1708 [28]	2.0	1.02
34	β-selinene	1480	1489	1.1	1.24	1702	1717 [28]	1.9	0.41
35	γ-muurolene	1484	1478	0.3	0.24	1678	1690 [28]	0.4	0.20
36	α-amorphene	1487	1483	0.6	0.20	1713	1710 [32]	0.8	0.25
37	viridiflorene	1491	1496	0.8	0.46	1684	1696 [28]	1.0	0.46
38	*epi*-cubebol	1503	1493	0.9	1.61	1880	1900 [28]	0.6	0.36
39	*trans*-calamenene	1510	1521	15.9	4.30	1821	1823 [28]	14.6	2.19
40	*trans*-cadina-1,4-diene	1520	1533	3.5	0.78	1771	1797 [33]	3.5	0.65
41	α-dehydro-*ar*-himachalene	1523	1516	0.2	0.05	1895	1882 [34]	0.2	0.04
42	α-calacorene	1527	1544	0.5	0.07	1903	1921 [28]	0.6	0.08
43	germacrene B	1541	1559	1.2	0.32	1811	1824 [28]	1.2	0.31
44	spathulenol	1565	1577	6.2	0.86	2117	2126 [28]	6.5	0.91
45	globulol	1576	1590	0.4	0.07	2066	2063 [35]	0.3	0.16
46	caryophyllene oxide	1592	1582	0.5	0.11	1966	1986 [28]	0.5	0.07
47	1-*epi*-cubenol	1627	1627	1.3	0.21	2048	2088 [28]	1.0	0.20
48	*epi-*α-cadinol	1630	1638	0.5	0.09	2163	2166 [36]	0.2	0.04
49	α-muurolol	1633	1644	0.5	0.08	2178	2183 [28]	0.5	0.08
50	cubenol	1641	1645	1.1	0.20	2055	2052 [37]	1.7	0.23
51	*cis*-calamenen-10-ol	1660	1660	0.3	0.07	2319	2315 [32]	0.7	0.10
	**Monoterpene hydrocarbons**			**13.9**				**13.7**	
	**Oxygenated monoterpene**			**15.0**				**13.6**	
	**Sesquiterpene hydrocarbons**			**55.1**				**57.2**	
	**Oxygenated sesquiterpene**			**11.7**				**12.0**	
	**Others**			**0.4**				**0.6**	
	**Total**			**96.1**				**97.1**	

LRI: calculated linear retention indices; LRI_r_: reference linear retention indices; ^1^: relative percentage amount; ^2^: standard deviation.

**Table 2:** Chemical composition of the essential oil of *M. mollis* in DB-5ms and HP-INNOWax columns.

N.	Compound	LRI	DB5-ms LRI_r_ [27]	%^1^	σ^2^	LRI	HP-INNOWax LRI_r_	%^1^	σ^2^
1	α-thujene	924	924	0.4	0.02	1022	1027 [28]	0.4	0.05
2	α-pinene	931	932	29.2	1.65	1017	1025 [28]	27.7	0.72
3	camphene	948	946	0.5	0.12	1060	1069 [28]	0.4	0.08
4	β-pinene	976	974	31.3	1.67	1106	1110 [28]	30.0	1.34
5	myrcene	988	988	5.0	2.73	1164	1161 [28]	5.2	2.81
6	α-terpinene	1005	1014	0.1	0.04	1176	1178 [28]	0.3	0.02
7	*p*-cymene	1015	1020	0.3	0.04	1269	1270 [28]	0.5	0.12
8	*o*-cymene	1023	1022	0.5	0.12	-	-	-	-
9	limonene	1027	1024	4.7	0.23	1197	1198 [28]	4.6	0.25
10	1.8-cineole	1031	1026	8.5	3.62	1203	1211 [28]	8.7	3.77
11	(*E*)-β-ocimene	1045	1044	0.3	0.03	1252	1250 [28]	0.4	0.03
12	γ-terpinene	1056	1054	1.4	0.14	1243	1245 [28]	1.5	0.19
13	terpinolene	1083	1086	0.7	0.15	1280	1282 [29]	0.8	0.18
14	linalool	1099	1095	7.7	2.42	1552	1554 [29]	8.2	2.71
15	α-ylangene	1373	1373	0.9	0.14	1481	1484 [28]	1.1	0.10
16	(*E*)-β-caryophyllene	1416	1417	2.3	0.18	1585	1599 [28]	2.7	0.03
17	germacrene D	1472	1480	0.4	0.04	1705	1708 [28]	0.3	0.28
18	δ-cadinene	1515	1513	1.2	0.14	1750	1756 [28]	2.1	0.09
	**Monoterpene hydrocarbons**			**74.4**				**71.8**	
	**Oxygenated monoterpene**			**16.2**				**16.9**	
	**Sesquiterpene hydrocarbons**			**4.8**				**6.2**	
	**Total**			**95.4**				**94.9**	

LRI: calculated linear retention indices; LRI_r_: reference linear retention indices; ^1^: relative percentage amount; ^2^: standard deviation.

**Table 3:** Enantioselective GC analysis of *M. myrsinoides* EO.

Enantiomers	Diethyl terbutylsilyl-β-cyclodextrin			Diacetyl terbutylsilyl-β-cyclodextrin		
	LRIs	Enantiomeric Distribution (%)	ee ^1^ (%)	LRIs	Enantiomeric Distribution (%)	ee ^1^ (%)
(+)-α-thujene	931	72.6	45.2	-	-	-
(-)-α-thujene	935	27.4		-	-	-
(*S*)-α-pinene	941	37.5	25.0	-	-	-
(*R*)-α-pinene	942	62.5		-	-	-
(+)-β-pinene	962	39.3	20.8	1018	40.3	19.4
(-)-β-pinene	968	60.1		988	59.7	
(+)-sabinene	990	36.9	26.2	1009	63.8	27.6
(-)-sabinene	1067	63.1		1013	36.2	
(+)-limonene	1062	100.0	100.0	-	-	-
(*R*)-α-phellandrene	-	-	-	1073	1.9	96.2
(*S*)-α-phellandrene	-	-	-	1028	98.1	
(*S*)-4-terpineol	1270	33.8	32.4	1376	63.2	26.4
(*R*)-4-terpineol	1273	66.2		1338	36.8	
(+)-germacrene D	1471	100.0	100.0	-	-	-

^1^: enantiomeric excess.

**Table 4:** Enantioselective GC analysis of *M. mollis* EO.

Enantiomers	Diethyl terbutylsilyl-β-cyclodextrin			Diacetyl terbutylsilyl-β-cyclodextrin		
	LRIs	Enantiomeric Distribution (%)	ee ^1^ (%)	LRIs	Enantiomeric Distribution (%)	ee ^1^ (%)
(+)-α-thujene	924	31.1	37.8	-	-	-
(-)-α-thujene	928	68.9		-	-	-
(*S*)-α-pinene	933	100.0	100.0	-	-	-
(+)-β-pinene	962	2.9	94.2	1013	0.2	99.6
(-)-β-pinene	968	97.1		982	99.8	
(*S*)-limonene	1062	94.6	89.2	-	-	-
(*R*)-limonene	1078	5.4		-	-	-
(+)-germacrene D	1477	100.0	100.0	-	-	-

^1^: enantiomeric excess.

**Table 5:** Active-olfactory compounds of the essential oils from *M. myrsinoides* and *M. mollis*.

LRIcal ^1^	LRIcal ^2^	Compound	AEDA (FD) ^1^	AEDA (FD) ^2^	Odor
919	-	α-pinene	8	-	woody
965	976	β-pinene	8	8	woody
1024	-	limonene	16	-	citrus
-	1031	1,8-cineole	-	8	minty
1051	1056	γ-terpinene	2	4	herbaceous
1079	1083	terpinolene	4	4	plastic
1100	1193	linalool	8	2	floral
1380	-	β-elemene	2	-	herbal
-	1416	(*E*)-β-caryophyllene	-	1	woody
1565	-	spathulenol	2	-	herbal

^1^: *M. myrsinoides* EO; ^2^: *M. mollis* EO.
